# Obesity and Air Pollution: Global Risk Factors for Pediatric Non-alcoholic Fatty Liver Disease

**DOI:** 10.5812/kowsar.1735143X.746

**Published:** 2011-10-01

**Authors:** Roya Kelishadi, Parinaz Poursafa

**Affiliations:** 1 Pediatrics Department, Child Health Promotion Research Center, Isfahan University of Medical Sciences, Isfahan, IR Iran; 2 Pediatrics Department, Faculty of Medicine, Isfahan University of Medical Sciences, Isfahan, IR Iran; 3 Department of Environment and Energy, Science and Research Branch, Islamic Azad University, Tehran, IR Iran; 4 Environment Research Center, Isfahan University of Medical Sciences, Isfahan, IR Iran

**Keywords:** Fatty Liver, Child, Obesity, Environmental Exposure, Prevention and Control, Air Pollution

## Abstract

Non-alcoholic fatty liver disease (NAFLD) is becoming as an important health problem in the pediatric age group. In addition to the well-documented role of obesity on the fatty changes in liver, there is a growing body of evidence about the role of environmental factors, such as smoking and air pollution, in NAFLD. Given that excess body fat and exposure to air pollutants is accompanied by systemic low-grade inflammation, oxidative stress, as well as alterations in insulin/insulin-like growth factor and insulin resistance, all of which are etiological factors related to NAFLD, an escalating trend in the incidence of pediatric NAFLD can be expected in the near future. This review focuses on the current knowledge regarding the epidemiology, diagnosis and pathogenesis of pediatric NAFLD. The review also highlights the importance of studying the underlying mechanisms of pediatric NAFLD and the need for broadening efforts in prevention and control of the main risk factors. The two main universal risk factors for N  LD, obesity and air pollution, have broad adverse health effects, and reducing their prevalence will help abate the serious health problems associated with pediatric NAFLD.

## 1. Introduction

Non-alcoholic fatty liver disease (NAFLD) is considered the most common liver disease in various age groups. Its development is strongly linked to obesity [1], as well as to the relative changes in body mass index in each individual, which may be related to the onset of fatty liver [2]. Even though liver steatosis has various causes in the pediatric age group, such as inherited metabolic disorders, malnutrition, infections, and drug toxicity, fatty liver disease is often seen in children in the absence of an apparent inherited metabolic defect or a specific cause. The vast majority of children with fatty liver disease are found to be obese and insulin resistant [1][2]. Low- and middle-income countries face the double burden of nutritional disorders, with an increasing prevalence of childhood obesity [3], and therefore, an increasing number of reports of NAFLD in the pediatric age group [4][5][6][7]. An increasing number of studies have proposed an association between environmental factors, namely air pollution, and fatty changes in the liver. This review will focus on the current knowledge regarding the epidemiology,diagnosis, and pathogenesis of pediatric NAFLD, as well as the possible associations with obesity and air pollution, which are the adverse effects of urbanization and globalization of lifestyle.

## 2. Global Trends in Childhood Obesity

The World Health Organization states “An escalating global epidemic of overweight and obesity– “globesity”– is taking over many parts of the world” [8]. Of special concern in the context of this epidemic is the escalating trend in the prevalence of childhood overweight and obesity on a global scale. There are several reports on the increasing prevalence of childhood obesity in industrialized countries [9][10][11][12][13][14]; however, this is an emerging health problem in low- and middle-income countries as well [15][16][17][18]. An analysis of 450 nationally representative crosssectional surveys of preschool-aged children from 144 countries indicated that in 2010, 43 million children, 35 million of them in developing countries, were estimated to be overweight and obese, and 92 million were at risk of becoming overweight. The global prevalence of childhood overweight and obesity increased from 4.2% (95% CI: 3.2%, 5.2%) in 1990 to 6.7% (95% CI: 5.6%, 7.7%) in 2010. This trend is expected to reach 9.1% (95% CI: 7.3%, 10.9%), or ≈60 mill  n, in 2020 [19]. It is noteworthy that in many cases, the excess weight of children in developing countries is because of their stunting [15][20][21]. These findings highlight the need for determining the barriers to healthy lifestyle [22] and promoting healthy living in their current obesogenic environments to reverse the anticipated health and social consequences of childhood overweight, namely NAFLD.

## 3. Histological Appearance of Pediatric NAFLD

The spectrum of NAFLD ranges from pure fatty infiltration (steatosis) to inflammation non-alcoholic steatohepatitis (NASH), fibrosis, and cirrhosis [23]. It accounts for up to 20% of abnormal liver function test results in most developed countries [24]. The histological appearance of NAFLD differs significantly in children and adults; it might represent a physiological response to environmental factors in children and a long-standing adaptation in adults. The histological criteria for distinguishing between adult (type 1) and pediatric (type 2) NASH have been proposed. Prominently, the histological features of liver injury seem to be associated with gender- and agespecific prevalence, i.e., type 2 NASH is more prevalent in younger children, and significantly more boys are affected by type 2 NASH than girls [25]. Among obese children, the severity of steatosis is found to be associated with increased visceral fat mass, insulin resistance, lower adiponectin levels, and higher blood pressure [26].

## 4. Diagnosis of Pediatric NAFLD

### 4.1. Biochemical Tests

Liver biopsy is the gold standard for diagnosis, but given that it is not feasible in large epidemiological studies, surrogate markers such as serum alanine/aspartate aminotransferases (ALT/AST) or ultrasonography are usually used to detect NAFLD [27]. The normal range of ALT/AST levels varies widely, and biopsy-proven NAFLD has been found in children with normal aminotransferase levels [25][28][29]. Aminotransferases, including aspartate AST and ALT, are commonly used in evaluating liver pathologies such as NAFLD and hepatitis. Given that AST is produced in different tissues such as the liver, heart, muscle, kidney, and brain, ALT has been generally accepted as a better predictor of liver injury. Usually in a clinical setting, an ALT level of 40 IU/L is considered the upper limit of the normal range [30]. However, some studies suggested lower cutoff values in children than in adults [31][32]. Moreover, some researchers have proposed gender differences for these levels, i.e., 19U/L and 30U/L for girls and boys, respectively [33][34].

### 4.2. Radiologic Diagnosis

The image-based diagnosis of NAFLD is usually straightforward, but fat accumulation may be manifested with unusual structural patterns that simulate other conditions. Fat deposition in the liver may be identified non-invasively with ultrasonography, computerized tomography, or magnetic resonance imaging [35][36]. In ultrasonography, the echogenicity of the normal liver nearly equals or slightly exceeds that of the renal cortex or spleen. Intrahepatic vessels are tightly defined, and the posterior parts of the liver are well-illustrated. Fatty liver may be identified if liver echogenicity exceeds that of the renal cortex and spleen, with attenuation of the ultrasound wave, loss of delineation of the diaphragm, and poor demarcation of the intrahepatic architecture [37][38].

## 5. Prevalence of Pediatric NAFLD

Determination of the prevalence of NAFLD accurately in children is difficult. Because of the aforementioned limitations and controversies in the diagnosis of NAFLD in children and adolescents, data based on surrogate markers might underestimate or overestimate the current burden of pediatric NAFLD. One of the strongest population-based studies, using the histologic definition for NAFLD, was conducted as a retrospective review of autopsies, performed from 1993 to 2003 on 742 children aged 2 to 19 years. The prevalence of NAFLD was estimated as 9.6%, ranging from 0.7% in children aged 2–4 years, to 17.3% in those aged 15–19 years, with the highest documented rate, as high as 38%, in obese children. It is of note that this study revealed differences in terms of race and ethnicity in the prevalence of pediatric NAFLD, with a prevalence of 11.8% in Hispanics, 10.2% in Asians, 8.6% in Whites, and 1.5% in Blacks [39]. Results from the US National Health and Nutrition Examination Survey (NHANES 1999–2004) reported a prevalence of 8% for NAFLD in adolescents, based on elevated serum ALT [40]. This prevalence is reported to be much higher among obese children and adolescents, ranging from 10% to 25% based on elevated ALT, compared with 42% to 77% based on ultrasonography [41][42][43][44]. Table provides a summary of prevalence studies on pediatric NAFLD [25][26][39][40][45][46][47][48][49][50][51][52][53][54][55][56][57][58][59][60][61][62][63].

**Table:** Summary of Studies on the Prevalence of Pediatric Non-alcoholic Fatty Liver Disease

	**Location**	**Population Studied**	**Aims**	**Findings**
Widhalm et al. (2010) [63]	Review	Review article	To provide a detailed review for diagnosis and management of NAFLD a and NASH a	The prevalence ranges from at least 3% in children overall to about 50% in obese children
Liu et al. (2010) [53]	China	231obese children and 24 non-obese children as controls	To compare biochemical indicators and carotid intima-media thickness (IMT)	The NAFLD group had greater carotid IMT, hyperlipidemia and hypertension than other groups. IMT correlated with BMI, NAFLD and ALT a
Lin et al. (2010) [52]	Taiwan	69 obese children aged 6-17 y	To identify biomarkers for liver steatosis in obese children	Thirty-eight (55.1%) subjects had liver steatosis, with elevated ALT in 27 (71.1%) of them
Caserta et al. (2010) [47]	Italy	642 adolescents aged 11-13 y	To determine the prevalence of NAFLD	NAFLD was found in 12.5% of participants, increasing to 23.0% in overweight ones. Increased IMT wasassociated with NAFLD
Nobili et al. (2010) {%545]	Italy	118 children with biopsy-proven NAFLD	To assess the association of severity of liver injury and lipid profile	The NAFLD activity and fibrosis scores had positive correlation with triglyceride/HDL, total cholesterol/HDL, and LDL/HDL ratios
Patton et al. (2010) [56]	USA	254 children aged 6-17 y	To determine the association of metabolic syndrome with NAFLD	65 (26%) had metabolic syndrome with greatest risk among those with severe steatosis; hepatocellular ballooning was associated with metabolic syndrome
Shi et al. (2009) [60]	China	308 obese children aged 9 to 14 y	To determine the prevalence of NAFLD and metabolic syndrome	Among all the obese children, the prevalence of NAFLD, NASH and metabolic syndrome was 65.9% , 20.5% and 24.7% respectively
Koebnick et al. (2009) [51]	USA	Hospitalized with NAFLD or obesity in 6-25 y	To investigate trends of NAFLD and obesity among hospitalized patients	Between 1986 to 1988 and 2004 to 2006, hospitalization increased from 0.9 to 4.3/100,000 for NAFLD, and from 35.5 to 114.7/100,000 for obesity
Reinehr et al. (2009) [57]	Germany	Obese children followed for 1 y	To determine the course of obesity associated NAFLD	20.6% of obese children had hypertension, 22.3% had dyslipidemia, 4.9% had impaired fasting glucose , and 29.3% had NAFLD
Denzer et al. (2009) [26]	Germany	532 obese subjects aged 8–19 y	To examine the prevalence and markers associated with NAFLD	Hepatic steatosis was higher in boys (41.1%) than in girls (17.2%) and was highest in postpubertal boys (51.2%) and lowest in postpubertal girls (12.2%)
Sharp et al. (2009) [59]	U.S.-Mexico border	31 patients aged 8-18 y	To describe the physical and metabolic characteristics of children diagnosed with NAFLD	The majority of cases were adolescents (12-17 y) and Mexican American. All subjects were overweight
Fu et al. (2009) [48]	Taiwan	220 students (97normal, 48overweight,75obese) 12y	To investigate the risk factors for NAFLD among adolescents	NAFLD was detected in 39.8% in total, 16.0% in normal ,50.5% in overweight, and 63.5% among obese adolescents
Rocha et al. (2009) [58]	Brazil	1801 children aged 11 to 18 y	To evaluate the prevalence and clinical characteristics of NAFLD	The prevalence of NAFLD was 2.3%, most of whom were male and white. Insulin resistance (IR) was observed in 22.9% of them
Graham et al. (2009) [49]	US A	Sample of 12-19 y from the NHANES1999 to 2002	To determine the association of metabolic syndrome and NAFLD	The metabolic syndrome was associated with ALT > 40 U/L (OR = 16.7, CI 6.2-45.1)
Carter-Kent et al. (2009) [46]	USA	130 children with biopsy-proven NAFLD	To assess clinical and laboratory predictors of NAFLD severity	Fibrosis was present in 87% of patients; of these, stage 3 (bridging fibrosis) was present in 20%
Alavian et al. (2009) [45]	Iran	966 children aged 7-18 y	To investigate the prevalence of NAFLD	Fatty liver was diagnosed by ultrasound in 7.1% of children. The prevalence of elevated ALT was 1.8%
Kelishadi et al. (2009) [50]	Iran	1107 children aged 6-18 y	To compare the prevalence of NAFLD in different BMI categories	Elevated ALT was documented in respectively 4.1of normal weight, 9.5%in overweight and 16.9% in obese group, respectively
Fraser et al. (2007) [40]	USA	NHANES participants, aged 12-19 y (1999–2004)	To determine the prevalence of NAFLD	a prevalence of NAFLD of 8% based on elevated ALT
Schwimmer et al. (2006) [39]	USA	742 children aged 2-19 y with autopsy	To determine the prevalence of biopsy-proven NAFLD	Fatty liver was present in 13% of subjects. ranging from 0.7% for ages 2 to 4 up to 17.3% for ages 15 to 19 y
Schwimmer et al. (2005) [25]	USA	127 obese 12th-grade students	To determine the prevalence of NAFLD	Unexplained ALT elevation was present in 23% of participants , in boys (44%) and in girls (7%)
Park et al. (2005) [55]	Korea	1594 children aged 10-19 y	To investigated the relation of NAFLD and the metabolic syndrome	The prevalence of elevated ALT (> 40 U/L) was 3.6% in boys and 2.8% in girls. The prevalence of metabolic syndrome was 3.3% in both boys and girls
Strauss et al. (2000)[61]	USA	2450 children aged 12-18 y	To determine the prevalence of NAFLD in different BMI categories	6% of overweight adolescents had elevated ALT levels; about 1% of obese adolescents had ALT levels over twice normal
Tominaga et al. (1995) [62]	Japan	810 students, ages 4-12 y	To determine the prevalence of NAFLD	The overall prevalence of NAFLD was 2.6%., boys (3.4%) and girls (1.8%), (P = 0.15)
Sharp et al. (2009) [56]	USA-Mexico	31 patients aged 8-18 y	To describe the characteristics of children diagnosed with NAFLD	The majority of children were aged 12-17 y and Mexican American. All subjects were overweight

^a^ Abbreviations: ALT, alanine aminotransferase; NAFLD; non-alcoholic fatty liver disease; NASH, nonalcoholic steatohepatitis

## 6. NAFLD or MAFLD?

Because of the well-documented interrelationships between the risk factors, metabolic alterations, and liver histology of NAFLD and metabolic syndrome, a recent review suggested the term MAFLD (metabolic syndromeassociated fatty liver disease), which might describe both groups of patients with common pathophysiological features more accurately [64]. A growing body of evidence proposes that NAFLD and metabolic syndrome are interrelated even from childhood. Many studies revealed that the components of the metabolic syndrome are strong predictors of increased ALT activity in NAFLD among children and adolescents [42][65][66][67][68][69][70][71]. It is also documented that the higher levels of components of metabolic syndrome increase the risk of elevated ALT or AST in children and adolescents [50].

## 7. Pediatric NAFLD and Early Atherosclerosis

NAFLD shares the same causal factors with metabolic syndrome, which are also major cardiovascular risk factors. While there are conflicting results about the association of NAFLD with atherosclerotic cardiovascular diseases [72], a review of some studies confirmed the proatherogenic role of NAFLD, and suggested that among adult populations it can be an independent risk factor for atherosclerotic cardiovascular diseases [73]. How-ever, a review of some other studies suggested that in spite of the existing association of NAFLD with the early onset of the metabolic and vascular pathogenic changes of atherosclerosis, the evidence for the relationship between NAFLD and cardiovascular diseases is weak [74]. A population-based cohort study of adults, aged 30–70 years, showed that the carotid-intima media thickness (C-IMT) values were strongly correlated with metabolic syndrome factors. No significant difference in C-IMT was found between patients with isolated NAFLD and in controls, whereas in patients with NA FLD associated with metabolic syndrome, the C-IMT values were significantly higher than those in patients with NAFLD alone. This study revealed a possible independent role of NAFLD in determining arterial stiffness, assessed by measuring the values of carotid-femoral pulse wave velocity [75]. Recent studies of children and adolescents confirmed the association of NAFLD with C-IMT, and suggested that the liver and blood vessels share common mediators [47][50][76][77]. The clinical importance of the associations of NAFLD with C-IMT in children and adolescents need to be confirmed through longitudinal studies.

## 8. Dietary and Physical Activity Habits Related to Pediatric NAFLD

There is a growing body of evidence about the significance of environmental background in the establishment and development of NAFLD from the early years of life. Unhealthy dietary habits, such as disproportionately high consumption of saturated fats and refined sugars, may harm adipose tissue architecture and homeostasis. They may also alter the peripheral and hepatic resistance to insulin-stimulated glucose uptake, thus favoring chronic low-grade inflammation. Excess nutrientsthat cannot be stored in adipose tissue would overflow to muscle tissue and the liver. Fat deposition in both sites increases insulin resistance and promotes further fat deposition [78][79]. Lifestyle, notably dietary habits, is associated with the development of NAFLD [80]. The diet most recommended for prevention and control of NAFLD is a low-carbohydrate diet, with a very limited amount of refined carbohydrates [81][82]. In our study of adolescents aged 12–18 years we found significant associations between insulin resistan and NAFLD, and similar risk factors and protective factors for these 2 interrelated disorders. Waist circumference and the ratio of apolipoprotein B to apolipoprotein A-I (ApoB/ApoA-I ratio) had the highest odds ratio (OR) in increasing the risk of insulin resistance and NAFLD, whereas cardiorespiratory fitness, followed by healthy eating index, decreased this risk significantly [50].

## 9. Environmental Factors Related to NAFLD

### 9.1. Smoking and NAFLD

A growing body of evidence supports the potential effects of exposure to some environmental factors on liver diseases. Environmental exposure related to toxic waste sites was associated with an increased prevalence of autoimmune liver disease [83][84]. Therefore, increasing attention is being given to the effects of environmental factors on liver diseases, including NAFLD. Many recent studies have also documented the association of smoking with the incidence of and acceleration of disease progression in NAFLD, as well as with advanced fibrosis in this process [85][86][87][88][89].

### 9.2. Air Pollution and NAFLD

The harmful effects of air pollutants on atherosclerotic cardiovascular diseases are well-documented [88]. These effects might be mediated through oxidative stress and insulin resistance [90], which are also known to have pivotal roles in the pathogenesis of fatty liver [91]. Hence, it can be assumed that such environmental factors might be also associated with NAFLD. It is well-documented that diesel exhaust particles (DEP), which are major constituents of atmospheric particulate matters (PM) in urban areas, generate reactive oxygen species (ROS) [92]. The ROS are generated via enzymatic reactions catalyzed by cytochrome P-450 [93], or by a non-enzymatic route [94]. In 2007, two experimental studies examined the effects of exposure to DEP on fatty liver for the first time. One of these studies revealed that exposure to DEP might increase oxidative stress, with concomitant aggravation of fatty changes in the livers of diabetic obese mice. This exposure increases the AST and ALT levels, liver weigh , and the degree of fatty change of the liver, as ascertained histologically. This study suggested that ROS, lipid peroxides, or inflammatory cytokines produced in the lungs might reach the liver, or soluble constituents of PM might get transferred from the lung to the liver through systemic circulation. Given that exposure to these particles may decrease the mitochondrial membrane potential, and may increase ROS, followed by cytochrome-c release and inner mitochondrial membrane damage, this study proposed that mitochondrial damage could have an enhancing effect on NAFLD, especially in augmenting the effects of oxidative stress on the liver [95]. The other experimental study assessed the effects of oxidative stress elicited by DEP in the aorta, liver, and lungs of dyslipidemic ApoE(-/-) mice, at the age when visual plaques appeared in the aorta. Vascular effects secondary to pulmonary inflammation were omitted by injecting DEP into the peritoneum. Six hours later, the expression of inducible nitric oxidensy  hase (iNOS) mRNA increased in the liver. Injection of DEP did not induce inflammation or oxidative damage to DNA in the lungs and aorta. Therefore, the study proposed a direct effect of DEP on inflammation and oxidative damage to DNA in the liver of dyslipidemic mice [96]. Another study investigated the effects of a 6-weekexposure to filtered air, in comparison with ambient air PM at doses mimicking naturally occurring levels, on diet-induced hepatic steatosis in mice fed high-fat diets. Progression of NAFLD was evaluated by histologi cal examination of hepatic inflammation and fibrosis. This study showed that ambient PM reaches the liver by crossing the alveolar membranes and passing into circulation. Circulating fine PM may then accumulate in hepatic Kupffer cells, and has the potential to induce Kupffer cell cytokine secretion, which in turn triggers inflammation and collagen synthesis in hepatic stellate cells [97]. It is noteworthy that interleukin-6, the concentration of which increased up to 7-fold  in the abovementioned study, is also significantly abundant in cases of human NAFLD [98]. Some human studies confirmed the harmful effects of environmental toxins on liver diseases. For instance, it has been reported that non-obese chemical workers highly exposed to vinyl chloride may develop insulin resistance and toxicant-associated steatohepatitis [99]. Limited data exists on the potential role of environmental pollution on liver disease in the general population. A large population-based study was conducted on 4582 adult participants without viral hepatitis, hemochromatosis, or alcoholic liver disease, from the National Health and Nutrition Examination Survey (NHANES) in 2003-2004, to investigate whether environmental pollutants are associated with an elevation in serum ALT and suspected NAFLD. The ORs for ALT elevation were determined across exposure quartiles for 17 pollutants, after adjustments for age, race/ethnicity, sex, body mass index, poverty income ratio, and insulin resistance. It showed that exposure to polychlorinated biphenyls as well as heavy metals, notably lead and mercury, was associated with unexplained ALT elevation, and increased adjusted ORs for ALT elevation in a dose-dependent manner [100]. Given the susceptibility of children and adolescents to the harmful effects of air pollutants, including their effects on oxidative stress and insulin resistance documented even in moderate levels of air pollution [101], similar effects of air pollutants on pediatric NAFLD can be expected.

addition, a growing number of studies suggest that air pollution can aggravate the adverse effects of obesity and insulin resistance. As cited in the statements of the American Heart Association [86], our study among Iranian children and adolescents provided the first biological evidence for the association of air pollutant-induced systemic pro-inflammatory and oxidative responses with metabolic syndrome [101]. Similarly, a study in Canada revealed that long-term traffic exposure (NO2 level, by residence) was associated with a nearly 17% increase in the risk of having diabetes mellitus [102]. Similarly, some other studies have documented the association of exposure to air pollutants with metabolic syndrome, as well as susceptibility to diabetes mellitus and aggravation of its complications [103][104][105]. Given the inflammatory and oxidative properties of air pollutants, as well as their association with insulin resistance and metabolic syndrome, and considering the interaction of the latter cond tions with fatty changes in liver, more studies about the effects of environmental factors, notably air pollution, on NAFLD are warranted. The high susceptibility susceptibility of the pediatric age group to the harmful effects of air pollutants, especially pertaining to early stages of chronic diseases [22][50][106][107][108], further stresses that more attention should be given to preventing late-onset effects of air pollutants.

## 10. Conclusion

The prevalence of childhood obesity and air pollution is dramatically increasing on a global scale. Given that both excess body fat and exposure to air pollutants are accompanied by systemic low-grade inflammation, oxidative stress as well as alterations in insulin/insulin-like growth factor and insulin resistance, which contribute to fatty liver, an escalating trend in the incidence of pediatric NAFLD and its related complications can be expected in the near future. Studying the underlying mechanisms and broadening efforts to prevent and control the 2 main universal risk factors, obesity and air pollution, which have broad adverse health effects, will help abate the serious health problems associated with pediatric NAFLD.
